# Towards Personalised Therapy in Chronic Spontaneous Urticaria: Advancing From Endotype to Clinical Response

**DOI:** 10.1111/cea.70100

**Published:** 2025-07-02

**Authors:** Katie Ridge, Rizwan Ahmad, Barry Moran, Cliona O'Farrelly, Jean Dunne, Conor M. Finlay, Alan D. Irvine, Niall Conlon

**Affiliations:** ^1^ UCARE Centre, Clinical and Diagnostic Immunology St. James's Hospital Dublin Ireland; ^2^ School of Medicine, Trinity College Dublin Dublin Ireland; ^3^ School of Biochemistry and Immunology Trinity Biomedical Sciences Institute, Trinity College Dublin Dublin Ireland; ^4^ Trinity Translational Medicine Institute, School of Medicine, Trinity College Dublin Trinity Kidney Centre Dublin Ireland

**Keywords:** biomarkers, chronic spontaneous urticaria, dermatology, flow cytometry, mast cells, translational immunology

## Abstract

Chronic spontaneous urticaria (CSU) is a skin disorder characterised by recurrent hives and swellings that has a profound effect upon quality of life. Current guidelines for the management of CSU outline sequential use of standard dosing nonsedating H1 antihistamines, fourfold dose antihistamines and the anti‐IgE monoclonal antibody omalizumab. A proportion of patients will have partial response or no response to omalizumab despite uptitration of dose. Evidence suggests that nonresponders may represent a specific endotype of Type IIb autoimmune CSU and respond better to ciclosporin, a fourth line off‐licence treatment. Accurate and timely classification of CSU by endotype may enable personalised medicine for patients. Current attempts to classify CSU are based on distinct autoallergic and autoimmune pathways towards mast cell activation; Type I autoallergic urticaria as evidenced by IgE autoantibodies initiating FcεR1 crosslinking and Type IIb autoimmune urticaria as evidenced by IgG autoantibodies initiating FcεR1 crosslinking. However, recent data have demonstrated that the distinction between CSU endotypes is more nuanced, with overlap between categories whereby patients with Type IIb autoimmune CSU have been found to have coexistent IgE autoantibodies. A cohort of patients do not meet criteria for either endotype. Furthermore, there is recognition that laboratory parameters currently used to stratify patients are not widely available, hampering their practical use. This review seeks to summarise data on biomarkers associated with treatment response in CSU. While previous literature has focussed upon treatment response to antihistamines, our emphasis is on predicting treatment response to third and fourth‐line treatments, with further reference to emerging treatments that do not yet form part of guidelines for management of CSU. Understanding factors that influence clinical response to all agents is particularly important as treatment options for CSU rapidly expand. In the following sections, we will evaluate the biochemical and clinical parameters that have been explored in these patients as well as their potential utility in routine clinical practice.

## Graphical abstract

Chronic spontaneous urticaria (CSU) is a debilitating skin disorder with significant impact upon quality of life. Current guidelines advocate stepwise treatment of standard dose antihistamines, high dose antihistamines, omalizumab and ciclosporin. Endotype‐based classification may guide personalised therapy, but distinctions are nuanced and some patients may not fit defined categories. Accessible biomarkers predicting treatment response remain limited. This review examines how we might predict treatment response in CSU as disease management rapidly evolves.

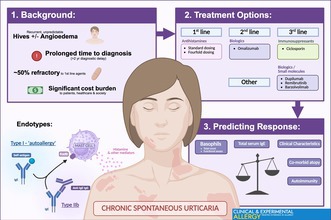

**Summary**

- Chronic spontaneous urticaria (CSU) is a heterogeneous disease.
- Endotype‐based classification is helpful, but distinctions are nuanced and some patients do not fit defined categories.
- Focusing on accessible biomarkers to predict response to current and emerging CSU treatments is essential.

## 1 Chronic Spontaneous Urticaria

Chronic spontaneous urticaria (CSU) is a skin condition characterised by recurring episodes of hives lasting longer than 6 weeks [1]. Angioedema, or swelling of the deeper layers of the skin, is a feature in approximately 50% of cases [2]. The characteristic hives seen in CSU are the result of endogenous signals and are not associated with specific allergic triggers.

A recent systematic review and meta‐analysis reports the overall lifetime prevalence of CSU as 1.4% (95% CI 0.8%–2.2%), with a point prevalence among adults of 0.86 (95% CI 0.12%–2.29%) and children (aged 0–19) of 1.43 (95% CI 0.89%–2.1%) [3]. CSU can affect any age group with a peak in the third to fifth decades [4, 5]. There is a female predominance in CSU that is more marked in adulthood and ranges from 70% to 78% [3, 6, 7].

The burden of CSU for patients and society is wide‐ranging. For individuals, the unpredictability of hives and swellings and potential resistance to treatment has a major impact upon quality of life and sleep with significant comorbidity of anxiety and depression among these patients [2, 6]. Time to accurate diagnosis and appropriate treatment may be prolonged, ranging from 2 to 4 years [2, 8, 9]. Productivity can be impaired and levels of absenteeism are comparable with moderate to severe psoriasis [10]. Unplanned healthcare attendances place strain upon healthcare services and as with many allergic diseases, the economic burden of CSU is likely underestimated [11]. CSU is a chronic condition with symptoms persisting for an average duration of 11.5 ± 10.8 years and more than half of patients having symptoms refractory to first‐line treatment [12, 13]. Omalizumab has demonstrated cost‐effectiveness in several healthcare systems but it is not effective for all patients. A range of emerging treatment options will require careful evaluation [14].

## 2 Pathophysiology of CSU

Mast cell degranulation is at the core of CSU pathogenesis, resulting in the release of preformed histamine and other vasoactive mediators such as leucotrienes, platelet activation factor and prostaglandin D2 [15, 16, 17]. These mediators induce endothelial cell activation resulting in increased vascular permeability and swelling of the deep layers of the skin [15, 18]. The events precipitating mast cell degranulation in CSU are likely multifactorial. Two autoreactive paradigms have been proposed to describe mast cell activation in CSU. The first, termed ‘autoallergy’ or Type I autoimmunity involves IgE binding to self‐antigen or ‘auto‐allergen’ in the skin [18]. The second, termed Type IIb autoimmunity involves IgG antibodies targeting IgE or the FcεR1 receptor [19, 20]. Both Type I autoallergy and Type IIb autoimmunity result in crosslinking of FcεR1 and mast cell degranulation. The release of mast cell mediators contributes not only to cell infiltration but also activation of the coagulation and complement cascades [21, 22]. While these distinct routes to mast cell degranulation have been posited to represent different endotypes of disease in CSU, overlap of mechanisms is apparent and the dichotomies presented may not offer a complete explanation of what is likely a heterogeneous condition [23, 24, 25].

Despite producing a similar clinical phenotype, differences in treatment response have been noted between patients with predominant Type I autoallergic or Type IIb autoimmune urticaria. Specifically, patients with biochemical evidence of autoimmune Type IIb urticaria, previously defined as the existence of a positive autologous serum skin test (ASST), the presence of IgG autoantibodies and positive basophil testing, show a better response to ciclosporin as opposed to anti‐IgE therapy [19, 20, 26, 27]. It is noteworthy that when these criteria are applied, only 8% of patients meet the criteria for autoimmune Type IIb urticaria [27]. Tests such as those detecting IgG autoantibodies to IgE or FcεR1 and the ASST are not widely available, even in specialist centres [19]. Furthermore, there is uncertainty about how the ASST might comply with blood product legislation in some jurisdictions.

In the emerging era of personalised medicine, early identification of the most effective treatment for an individual patient is attractive. While there has been a focus on nonresponse to high dose antihistamines in CSU, predicting treatment response to third and fourth‐line treatments and indeed newer treatments is less well understood [28]. Furthermore, identifying which patients may require increased doses or prolonged courses of omalizumab is an important consideration. In a meta‐analysis of 67 observational studies assessing clinical response to omalizumab, the average nonresponse rate was 7% with a partial response rate of 17.8% [29]. As treatment options for nonresponders to anti‐IgE treatments emerge, biomarkers that prospectively identify optimal treatments are essential. Here, we review the role of clinical and laboratory markers in predicting treatment response to currently available third and fourth‐line treatments as well as emerging treatments in CSU.

## 3 Methods

A search of the literature was conducted using electronic databases including PubMed, Embase and Google Scholar. The search used a combination of keywords and MeSH terms such as “chronic spontaneous urticaria,” “omalizumab,” “ciclosporin,” (and alternative spellings) “treatment response,” “clinical response”, “biomarkers” and “predictors.” Reference lists of key articles were also screened to identify additional relevant publications. Studies were included if they were published in English and addressed predictive factors for treatment response in CSU and included data on response to either omalizumab or ciclosporin or both. Both original research articles and relevant reviews published between 2005 and 2025 were considered. Articles that focused solely on treatment efficacy without discussing predictors or stratified outcomes were generally excluded unless they provided relevant subgroup analyses. We excluded articles that focussed on physical urticaria or urticarial vasculitis. A narrative review approach allowed for a broad overview of a rapidly evolving topic, while acknowledging the possibility of selection bias.

## 4 Biomarkers for Predicting Response to Anti‐IgE Treatment in CSU

### 4.1 Total Serum IgE

Three Phase III studies on the efficacy of omalizumab demonstrate that approximately a third of patients have a complete response to omalizumab, defined as a Urticaria Activity Score during a 7‐day period (UAS7) of 0 [30, 31, 32]. While early studies stated that baseline serum IgE was not predictive of clinical response, a re‐analysis of these data specifically interrogating treatment response in individuals with a low baseline serum IgE demonstrated that omalizumab had reduced efficacy in this subset of patients [33]. This finding has been replicated in other studies which suggest that a low serum IgE (typically < 18 IU/mL) correlates with a significantly reduced response to omalizumab [33, 34, 35]. Conversely, an elevated total serum IgE has been associated with complete response to omalizumab, defined as 90% improvement in symptom burden as evaluated by physicians’ global assessment after two doses [36]. Establishing a ‘cut‐off’ in total serum IgE to disentangle responders from nonresponders is a challenge and indeed, is not currently part of clinical guidelines for the management of CSU [1]. A recent evaluation of 158 patients receiving omalizumab found that the total baseline serum IgE ‘cut‐offs’ with the highest level of sensitivity (76.6%) and specificity (93%) for predicting complete response to omalizumab were 42.5 and 59.5 IU/mL, respectively [37] (See Table 1). The significance of serum circulating free IgE has been examined in CSU but has not been shown to predict response to omalizumab [38]. In conclusion, total serum IgE holds promise as a widely available and easily validated biomarker of treatment response, though establishing a standardised ‘cut‐off’ value remains a priority.

**TABLE 1:** Laboratory markers in CSU and their association with treatment response to omalizumab.

Baseline serum IgE and omalizumab treatment response		
Finding	References	Additional detail
Nonresponders to omalizumab have low baseline total IgE levels	[3]	Ertas: *n* = 113 Serum IgE measured prior to and after 3 months of omalizumab. CSU assessment: UAS7. Physicians and patients completed visual analogue scales (0–10) and treatment effectiveness scores (0–10). Nonresponders = No reduction in UAS7 or physical visual analogue scale or had severe symptoms after omalizumab. Partial responders = reduced disease activity but not complete remission (UAS7 > 1 or physician analogue scale > 1). Complete responders = UAS7 of 0 and physician analogue scale of 0 Straesser: *n* = 137 Serum IgE assessed prior to treatment. Clinical response 4 months later. CSU assessment: Subjective using prescriber's documentation +/− continuation of omalizumab.
High baseline serum IgE is associated with a complete response to omalizumab	[36]	Weller: *n* = 85 Serum IgE measured prior to and after 2 months of omalizumab. CSU assessment: Complete, partial and nonresponses were defined as reduction in signs and symptoms by > 90%, by 30%–90% and < 30%, respectively, using physician's global assessment
IgE ‘cut off’ that predicts complete response to omalizumab is 42.5–59.5 IU/mL	[37]	Ensina: *n* = 158 Serum IgE assessed prior to treatment. CSU assessment: UAS7 at baseline and 24 weeks later (after 6 months of omalizumab)
**Autoimmunity and omalizumab treatment response**		
Positive ASST is associated with a slower response to omalizumab	[39, 40]	Chen: *n* = 138 ASST assessed prior to omalizumab. CSU assessment: UCT after 3 months of omalizumab. Palladino: *n* = 15 ASST assessed prior to omalizumab CSU assessment: UAS7 after 1 month and 3 months of omalizumab. Early responders = Decrease in UAS7 < 16 after 1 month of treatment Late responders = Decrease in UAS7 < 16 only after 3 months of treatment
IgG autoantibodies are associated with slower response to omalizumab	[41, 42]	Niwa: *n* = 61 IgG anti‐IgE autoantibodies assessed using enzyme linked immunosorbent assay prior to commencement of omalizumab. CSU assessment: UAS7 at 1, 2 and 3 months. Late responders = UAS7 of ≤ 6 after Month 3 Kolkhir: *n* = 45 Antinuclear antibodies (ANA) and IgG anti‐TPO assessed prior to initiation of omalizumab CSU assessment: UAS7, Time 2 not explicitly stated Nonresponders = reduction in UAS7 of less than 30% Partial responders = reduction in UAS7 of 30%–89% Complete responders = reduction in 90% from baseline UAS7
Coexistence of IgG autoantibodies and IgE autoantibodies is associated with late or nonresponse	[25]	Maronese: *n* = 18 IgG anti‐FcεRI and IgE anti‐FcεRI quantified using enzyme linked immunosorbent assay prior to omalizumab CSU assessment: UAS7 every 4 weeks for 24 weeks (6 months of omalizumab). Early responders = UAS7 < 6 after 1 month Late responders = UAS7 < 6 between Month 1 and Month 3 Nonresponders = No change 4 months after omalizumab
Comorbid autoimmune disease is associated with a switch from omalizumab to ciclosporin due to inefficacy of omalizumab	[43]	Pesque: *n* = 173 CSU assessment: UAS7 and UCT at baseline and 6 months Clinical remission = UCT ≥ 12 or UAS6 < 7 after 6 months
**Basophils and omalizumab treatment response**		
High baseline blood basophil count predicts response to standard dose of omalizumab	[44]	Zubiaga Fernandes: *n* = 89 Blood basophil count and blood eosinophil count
Basopenia predicts nonresponse	[45]	Rijavec: *n* = 43 Total serum IgE, absolute cell count of basophils, CD63 basophil activation, CD63 activation of donor basophils after stimulation with sera of CSU patients Peripheral basopenia defined as < than 1,700 cells/mL. CSU assessment: UAS7 Early complete responders = UAS7 ≤ 6 within 2 weeks Late complete responders = UAS7 ≤ 6 within 12 weeks Nonresponse = no improvement or minor improvement within 12 weeks
Lower CD63 basophil activation predicts nonresponse	[45]	Rijavec: *n* = 43 Total serum IgE, absolute cell count of basophils, CD63 basophil activation, CD63 activation of donor basophils after stimulation with sera of CSU patients CSU assessment: UAS7 Early complete responders = UAS7 ≤ 6 within 2 weeks Late complete responders = UAS7 ≤ 6 within 12 weeks Nonresponse = no improvement or minor improvement within 12 weeks
Basophils with lower IgE receptor mediated histamine degranulation have slower response	[46]	Johal: *n* = 18 Basophil histamine release assay, basophil activation test, basophil surface IgE, FcεRI quantification Early responders ≥ 50% symptom reduction between Day 6–Day 30 of treatment as determined by UAS7 Late responders = Participants who did not achieve ≥ 50% symptom reduction by Day 30 but did achieve it by Day 90
Basophils with release of > 10% cellular histamine were fast and complete responders	[46]	Johal: *n* = 18 Basophil histamine release assay, basophil activation test, basophil surface IgE, FcεRI quantification Early responders ≥ 50% symptom reduction between Day 6–Day 30 of treatment as determined by UAS7 Late responders = Participants who did not achieve ≥ 50% symptom reduction by Day 30 but did achieve it by Day 90
Low baseline FcεRI receptor expression on basophils predicts nonresponse	[47]	Deza: *n* = 47 FcεRI receptor expression on basophils via flow cytometry CSU assessment: UAS7 and UCT assessed after 6 months of omalizumab Response to therapy was defined as UAS7 ≤ 6 after 6 months or a 90% reduction in UAS7 after 6 months.
High baseline FcεRI receptor expression on basophils predicts response within 6 months	[47]	Deza: *n* = 47. FcεRI receptor expression on basophils via flow cytometry CSU assessment: UAS7 and UCT assessed after 6 months of omalizumab. Response to therapy was defined as UAS7 ≤ 6 after 6 months or a 90% reduction in UAS7 after 6 months
Positive BHRA predicts need for higher doses/shorter duration between doses/discontinuation due to inadequate effect	[48]	Baumann: *n* = 209 Basophil histamine release assay CSU assessment: UAS7 Patients were assessed during first 12 months of treatment
Positive BHRA suggests slow or nonresponse	[49]	Gericke: *n* = 46 CSU assessment: UAS7 Response to therapy was defined as UAS7 ≤ 6 Fast responders = UAS7 ≤ 6 within 8 days Slow responders = UAS7 ≤ 6 between 9 days–3 months Nonresponders = UAS7 ≤ 7 after 3 months
**Eosinophils and omalizumab treatment response**		
Eosinopenia is associated with the need for higher doses	[26]	Kolkhir: *n* = 1613 Eosinopenia defined as < 0.05 × 10^9^/L CSU assessment: UAS 7
Eosinopenia is associated with complete nonresponse to standard dose omalizumab	[48]	Baumann: *n* = 209 Eosinopenia defined as < 0.05 × 10^9^/L CSU assessment: UAS7 Patients were assessed during first 12 months of treatment
**Endothelial cell activation, fibrinolysis and omalizumab treatment response**		
High D‐dimer predicts prompt response	[50]	Asero: *n* = 74 D‐dimer prior to omalizumab therapy CSU assessment: UAS7 after 3 months of omalizumab Early responders = UAS7 < 14 within 3–7 days of first dose Late responders = UAS7 < 20 after 2 months of treatment Nonresponders = Symptoms unchanged after 3 months of omalizumab
High D‐dimer predicts need for updosing	[51]	Tuncay: *n* = 159 D‐dimer (cut‐off 0.46 mg/dL) at baseline CSU assessment: UCT at baseline and at 4 months Nonresponder = UCT score of < 12 points and an increase of < 3 compared to baseline Responder = UCT score of ≥ 12 points and an increase of ≥ 3 compared to baseline Those with scores < 12 points and an increase in < 3 compared to baseline are accepted as uncontrolled or nonresponder and those ≥ 12 points and an increase of ≥ 3 compared to baseline are accepted as controlled or responder
No relationship between D‐dimer and treatment response	[28, 52]	Fok: Systematic review Atik: *n* = 88 CSU assessment: UCT after 3 months of omalizumab Responder = UCT ≥ 12 at 3 months Nonresponder = UCT < 12 at 3 months

### 4.2 Autoimmunity

A 12‐centre international study with 182 participants sought to establish criteria for identifying type IIb autoimmune urticaria [27]. This study defined autoimmune Type IIb urticaria as requiring three positive laboratory test results: a positive ASST, a positive immunoassay indicating the presence of IgG anti‐FcεR1 antibodies or IgG anti‐IgE antibodies and a positive basophil test (basophil activation test (BAT) or basophil histamine release assay (BHRA)). Using these criteria, less than 10% of patients were identified as having Type IIb autoimmune urticaria. These patients were found to be more likely to have a lower baseline serum IgE, more severe disease as defined by higher Urticaria Activity Scores (UAS7) and higher levels of anti‐TPO antibodies [53]. Taking each of these laboratory tests in isolation, a positive ASST has been associated with slower response (as opposed to nonresponse) to omalizumab ranging from 1 to 3 months [39, 40] (See Table 1). Similarly, IgG anti‐IgE autoantibody and IgG anti‐FcεRIα autoantibody levels were significantly higher in individuals who did not respond to omalizumab within 4 weeks [41]. Basophil characteristics are discussed separately below. However, in brief, individuals with a positive BHRA have been found to be slower responders or nonresponders to standard dose omalizumab [49]. Serological evidence of autoimmunity, including a positive ANA test and the presence of IgG anti‐TPO antibodies have also been associated with a poorer response to omalizumab [42, 43].

Although patients with autoimmune Type IIb urticaria have traditionally been held in direct contrast with patients with autoallergic urticaria, characterised by IgE autoantibodies such as IgE anti‐TPO or IgE anti‐IL24, growing evidence suggests that overlap in autoantibody isotypes in patients with CSU may exist [23, 54]. In fact, coexistence of IgG autoantibodies with IgE autoantibodies predicted late or nonresponse to omalizumab [25]. The autoantibody status of early responders to omalizumab in this study (IgG autoantibodies, IgE autoantibodies or none) was not reported [25].

While it is likely that the tests used in CSU phenotyping have highlighted pertinent disease characteristics, their reliance on specialist assays represents a challenge to clinicians in routine clinical practice.

Recognition of a need to focus upon standard laboratory assays where possible has led to the evaluation of combining routine assays to better predict treatment response in CSU. A study investigating whether high levels of IgG anti‐TPO antibodies and low serum IgE could be used as surrogate markers of autoimmune type IIb CSU yielded encouraging results, whereby high IgG anti‐TPO combined with low serum IgE correlated with results of BAT, ASST, basopenia and eosinopenia. However, while this study assessed treatment response to standard dosing of antihistamines, correlation with response to omalizumab was not assessed [19]. There is acknowledgement of a requirement to better define the predictive values, sensitivity and specificity of using IgG anti‐TPO and serum IgE as biomarkers in CSU [55]. A machine learning approach that tested the possible utility of combining laboratory tests, identified a specific cluster most closely aligned to autoimmune Type IIb urticaria, which was characterised by rates of IgG anti‐TPO and ANA positivity [56].

### 4.3 Basophils and Eosinophils

Peripheral basopenia is a well‐documented phenomenon in CSU [57]. The migration of basophils towards the skin and the intravascular destruction of basophils via the presence of functional autoantibodies have been proposed as contributors towards peripheral basopenia [58, 59]. Eosinophils are thought to be recruited to urticarial lesions as part of Type 2 inflammation whereby mast cell‐related cytokines lead to late‐phase eosinophil infiltration [21]. It is intuitive therefore to expect that peripheral basopenia and eosinopenia would be markers of disease severity in CSU, and this is borne out in the literature [26, 60]. Furthermore, basophil counts have been found to increase with the resolution of CSU symptoms [61, 62].

A study of 89 patients reported that a higher baseline blood basophil count (20 cells/μL) was predictive of responding to a standard dose of omalizumab [44]. Similarly, individuals with a peripheral basopenia of less than 1700 cells/mL (or 1.7 cells per μL) and lower basophil CD63 activation were more likely to be omalizumab nonresponders [45]. It is important to consider, however, that routine measurement of basophils in peripheral blood may be a crude measure for assessing basophil activity in disease states. Automated methods for counting granulocytes have been found to have moderate accuracy only, and laboratory techniques for identifying basophils by flow cytometry are not standardised [63]. Functional basophil assays which interrogate basophil characteristics may provide us with important tools for predicting treatment response in CSU. In a study of basophil functional phenotype, patient basophils with lower IgE receptor‐mediated histamine degranulation demonstrated a slower treatment response to omalizumab [46]. Conversely, patients with basophils that demonstrated a release of > 10% of cellular histamine were more likely to be fast and complete responders to omalizumab [46]. Baseline FcεRI receptor expression on basophils was lower in individuals who did not respond and higher in those who did respond to 6 months of omalizumab therapy [47]. Patients with a positive basophil histamine release assay have also been found to require higher doses of omalizumab for disease control [48].

Studies have shown how eosinopenia in CSU is associated with both the need for higher doses of omalizumab and complete nonresponse [26, 48]. Kolkhir et al. report that eosinopenia is a correlate of other parameters traditionally associated with Type IIb autoimmune CSU including ASST positivity, BHRA positivity and low total serum IgE [26]. While, eosinophilic cationic protein has recently been found to be associated with response to antihistamines, this parameter was not associated with response to omalizumab [52].

### 4.4 Endothelial Cell Activation and Markers of Fibrinolysis

The activation of eosinophils in CSU engages the coagulation cascade via the expression of tissue factor [21]. In addition, histamine and proinflammatory mediators lead to activation of the endothelium. Enhanced endothelial cell activation and thrombin generation in patients with CSU is apparent [64]. Markers of fibrinolysis such as serum D‐dimer are well established as signifiers of disease activity in CSU and have furthermore been associated with nonresponse to antihistamine therapy [65]. Interrogation of the association between serum D‐dimer and clinical response to omalizumab has shown conflicting results. One study reports that an elevated D‐dimer is a marker of prompt response to omalizumab [50]. Others have found no relationship between D‐dimer and treatment response to omalizumab [28, 52]. A recent study reports that an elevated D‐dimer prior to initiation of omalizumab was associated with a 4.8‐fold increase in the requirement for updosing to 600 mg [51]. The exploration of endothelial cell activation in CSU represents an area for further exploration in relation to treatment response [66].

## 5 Predicting Response to Ciclosporin

**FIGURE 1:**
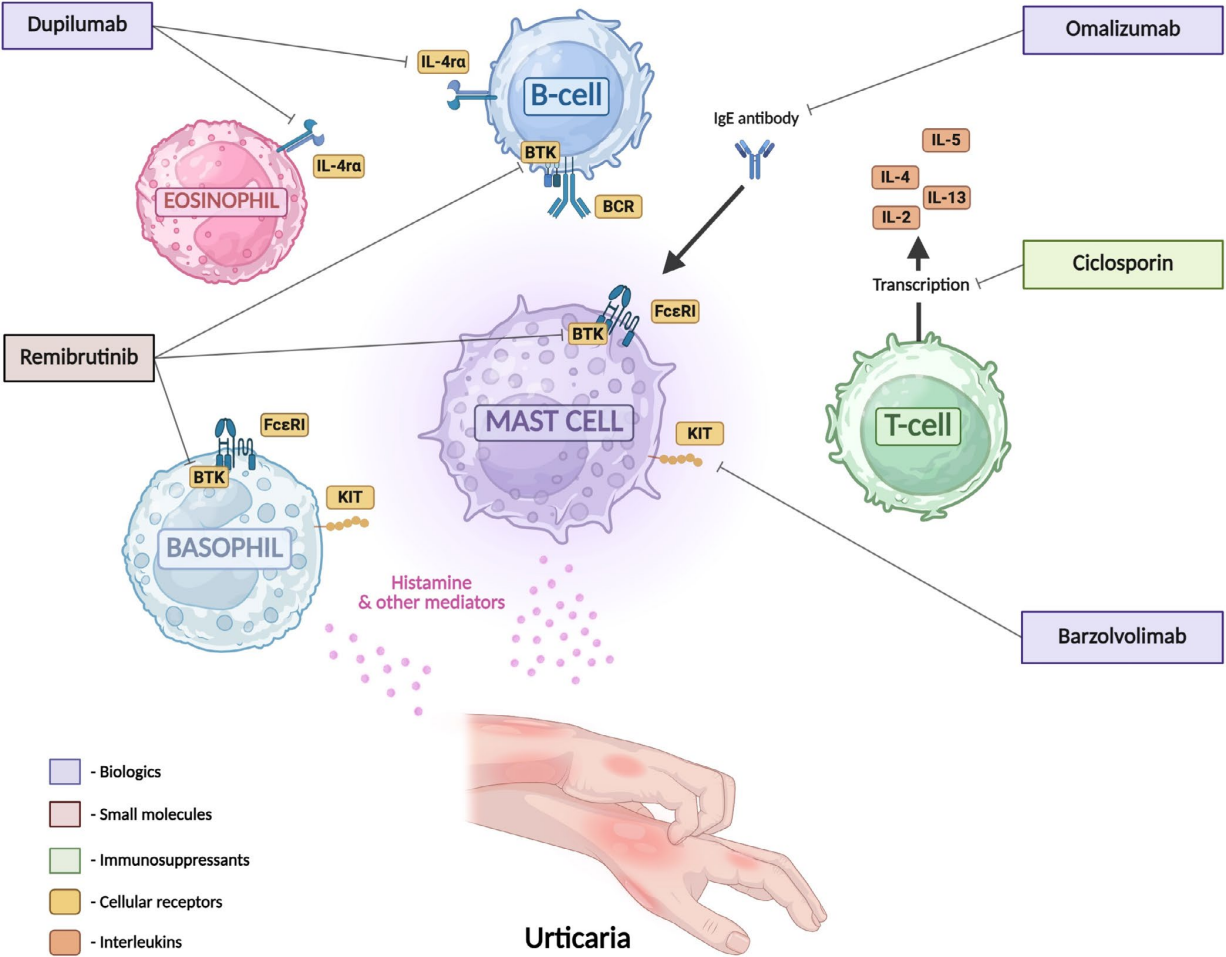
Treatment approaches in chronic spontaneous urticaria.

Ciclosporin at a dose of up to 5 mg/kg is recommended as a fourth‐line treatment for the management of CSU that is refractory to high dose omalizumab [1] (See Figure 1). While current treatment guidelines do not outline criteria for testing for autoimmune type IIb CSU, this group may be over‐represented among patients who are commenced on ciclosporin having exhausted all other treatment options. It is unsurprising, therefore, that laboratory tests such as a positive BHRA, which is closely aligned with autoimmune Type IIb CSU, have been identified as markers of response to ciclosporin [67, 68]. While one study reports no significant differences between patients who respond to omalizumab alone versus patients who respond to ciclosporin alone, another proposes that ciclosporin responders have higher baseline disease activity, low baseline IgE and normal baseline D‐Dimer levels [69] (See Table 2). A normal baseline D‐dimer was also found to be associated with complete response to ciclosporin in a prospective study of 29 patients receiving ciclosporin for CSU [70].

**TABLE 2:** Factors associated with response to ciclosporin.

Finding	References	Additional detail
Positive BHRA is associated with treatment response	[68]	Iqbal: *n* = 58 Basophil histamine release assay prior to treatment with ciclosporin CSU assessment: Assessment by attending clinician Immediate responders = response within days Early responders = response within 1 month Late responders = response between Month 1–Month 3
Higher baseline disease activity is associated with treatment response Low baseline IgE is associated with treatment response	[69]	Mehta: *n* = 132 Baseline UAS7, baseline IgE and D‐dimer Elevated IgE defined as > 100 IU/mL Treatment response was defined as > 90% recorded reduction in UAS7 after 4 months of treatment
Elevated baseline D‐dimer level associated with nonresponse	[69, 70]	Mehta: *n* = 132 Treatment response was defined as > 90% recorded reduction in UAS7 after 4 months of treatment Elevated D‐dimer defined as > 500 ng/mL Treatment response was defined as > 90% recorded reduction in UAS7 after 4 months of treatment Asero: *n* = 29 Elevated D‐dimer defined as > 500 ng/mL Clinical response was assigned by a clinician as no benefit (Score 0), partial benefit (Score 1), good benefit (50%–80% reduction) (Score 3), excellent benefit (complete absence of pruritius and wheals taking antihistamines at licensed dose) (Score 4) and remission while off antihistamines (Score 5).
Comorbid autoimmune disease is associated with a switch from omalizumab to ciclosporin due to inefficacy of omalizumab	[43]	Pesque: *n* = 173 CSU assessment: UAS7 and UCT at baseline and 6 months Clinical remission = UCT ≥ 12 or UAS6 < 7 after 6 months

## 6 Clinical Characteristics as a Predictor of Treatment Response

Although female sex has been identified as a negative predictor of therapy response in dermatological conditions overall, evidence for the impact of sex on treatment response in CSU is conflicting [71]. One study has demonstrated that males are more likely to require longer courses of omalizumab when compared with females, while another identifies females as having higher rates of recurrence [72, 73]. There is no reported association between age and response to omalizumab or ciclosporin [28]. Comorbid autoimmune disease has been highlighted as an important clinical characteristic which might raise suspicion of autoimmune Type IIb CSU and thereby nonresponse to omalizumab. In an observational study, patients with a reported comorbid autoimmune condition were more likely to have been switched from omalizumab to ciclosporin [43]. While the presence of angioedema has traditionally been appraised as a marker of disease severity, there is conflicting evidence as to its role in predicting treatment response [69, 74]. A study comparing responders and nonresponders to omalizumab and ciclosporin found that concomitant inducible urticaria and angioedema were associated with nonresponse to both treatments [69]. However, data from large multicentre studies report that the presence of angioedema did not predict nonresponse and that comorbid inducible urticaria is not associated with poorer response to omalizumab [75, 76]. There is some evidence to suggest that patients with depressive symptoms have a higher incidence of nonresponse to omalizumab than patients without [77]. Obesity has also been proposed as a predictor of nonresponse [78].

## 7 The Emergence of Novel Markers as Predictors of Response in CSU

A recent study on the marker Galectin 9, a widely expressed protein involved in immune regulation, describes how responders to three doses of omalizumab (achieving a UAS7 of > 16) had significantly higher baseline numbers of Galectin 9+ eosinophils. The study also evaluated Galectin 9+ basophils and while these cells were numerically higher in omalizumab responders, findings were not statistically significant. Confirmation of the relationship between Galectin 9 expression and how this is related to treatment response more broadly represents an area for further exploration in CSU [79]. Myeloid and mast cell progenitors in patients with CSU identified by flow cytometry predicted treatment response to omalizumab. These findings were confirmed using transcriptomic data [80]. A recent study on plasma proteomics in a group of 96 patients with CSU found that while changes in plasma proteins were associated with clinical characteristics, (e.g., metallopeptidase‐9 and thrombospondin relating to presence of angioedema) there was no significant association between plasma proteins and treatment response to omalizumab [81].

## 8 Emerging Therapies in CSU and Treatment Response

It is important to note that anti‐IgE biosimilars are in development [82]. The financial implications of this advancement have the potential to transform access to treatment in CSU. Beyond anti‐IgE treatments, additional agents that are not yet part of clinical guidelines have undergone significant evaluation, with the notable case of dupilumab, an IL‐4Rα blocker, in open‐label extension studies following Phase 3 trials. While head‐to‐head trials comparing the efficacy of newer agents with existing third and fourth‐line treatment options have not been completed, indirect evidence demonstrates the effectiveness of dupilumab, remibrutinib and barzolvolimab in CSU [83, 84, 85] (see Figure 1). Phase 2b trials for benralizumab in CSU, an anti‐IL‐5Rα monoclonal antibody, were discontinued as a result of no benefit derived from benralizumab versus placebo at 12 weeks [86].

## 9 Predicting Response to Anti‐IL4R Therapy

Evaluation of dupilumab included a cohort of individuals who either did not tolerate or had only partial response to omalizumab (*n* = 54). This trial was halted early. Nonetheless, UAS7 did improve after 24 weeks of dupilumab but findings were not statistically significant [83]. In contrast, individuals who were omalizumab naïve (*n* = 138) had a significant improvement in UAS7 scores after 24 weeks. Importantly, baseline serum IgE, did not predict treatment response to dupilumab [87]. A study of a small number of omalizumab nonresponders (*n* = 7) demonstrated complete response (UAS7 of 0) to dupilumab, with time to response varying between 1 and 6 months [88]. Time to response is an important consideration, as comparisons of the ‘time to first response’ in dupilumab and omalizumab reveal important differences. Omalizumab demonstrates more rapid clinical improvements which can lead to treatment plateau, whereas treatment benefit in dupilumab appears to increase incrementally [89].

As well as differences in time to response, dupilumab may offer distinct benefits for individuals with comorbid atopic disease. Due to its impedance of IL‐4/IL‐13, patients with CSU with comorbid atopic dermatitis or chronic rhinosinusitis with recurrent nasal polyposis may stand to benefit significantly from dupilumab therapy [90, 91]. Therefore, specific patient factors may be key in selecting this agent.

## 10 BTK Inhibitors

The evaluation of remibrutinib in CSU is in Phase 3 development and to date, has not specifically interrogated effectiveness in omalizumab nonresponders. Data on efficacy are nonetheless, robust, with a mean reduction in UAS7 scores at week 12 of −20 ± 7 versus placebo (−13.9 ± 1) [92]. Published results suggest no difference in treatment response in patients treated with remibrutinib who had previously received omalizumab therapy versus those who had not [84]. Furthermore, remibrutinib has been found to improve UAS7 in all patients when stratified by baseline IgE (≤ 43 IU/mL and > 44 IU/mL) [93]. While characteristics that predict favourable treatment response to remibrutinib have not been published, an in vitro study demonstrates that remibrutinib inhibits activation and degranulation of basophils and mast cells on exposure to sera of CSU patients [94]. Of note, this effect was observed on treatment of cells with sera of both omalizumab responders and nonresponders. Further work in this area will establish whether the modulation of intracellular signalling cascades targeted by remibrutinib, holds promise for patients with CSU that is recalcitrant to currently available therapies.

Use of the anti‐KIT monoclonal antibody barzolvolimab, has undergone Phase 2 testing with minimal data published at present [85]. It is worth noting however that through mast cell depletion, this agent could potentially mitigate against diverse pathways towards mast cell degranulation. As guidelines for the management of CSU are updated, it remains to be seen how emerging treatments will complement existing therapeutics.

## 11 Conclusion

Attempts to identify predictors of treatment response in CSU have expanded from basic clinical and laboratory parameters to the use of specialist autoreactivity assays and novel flow cytometry approaches. While there has been a focus on disease endotype, there is a dearth of trials evaluating and interrogating clinical response with respect to third and fourth‐line treatment choices in CSU. Although the utility of baseline IgE in predicting response to omalizumab remains debated, it is apparent that individuals with a very low serum IgE (< 18 IU/mL) tend to have poor clinical outcomes. Coexistence of IgG and IgE autoantibodies may indicate delayed or nonresponse to omalizumab, but complex and nonstandardised testing remains a challenge with regard to these assays. Functional basophil assays investigating histamine release and FcεRI expression may offer valuable insights over basophil counts, in evaluating omalizumab response. However, widespread utilisation of such assays may be hampered by sample requirements and assay availability. D‐dimer is a robust marker of disease activity in CSU; however, data on the association with omalizumab response are inconsistent. A vital question held by all who treat CSU is whether emerging therapies offer effective treatment options for omalizumab nonresponders. As of yet, strong, direct evidence on any emerging treatment that delivers efficacy for omalizumab nonresponders is awaited.

As the number of pharmacological options for patients with CSU increases, developing a practical approach to allow personalised treatment decisions is essential to improve outcomes and minimise medication costs. Importantly, any approach needs to be implementable as part of routine clinical care and should profit from expanding clinical and biochemical data. As echoed by other authors, there is a clear role for the Urticaria Centres of Reference and Excellence (UCARE) network to develop and utilise highly annotated biobanks of patients with CSU in order to inform clinical decision making [55]. Large, adequately powered studies leveraging artificial intelligence modalities should be employed to combine clinical, immunological and other variables to remove redundant parameters and tease out what factors are associated with response and nonresponse to current and new medications. Despite significant interest, CSU endotyping has not yet translated into routine clinical practice as far as medication selection is concerned. Increased understanding of the nuance of disease pathogenesis and treatment outcomes in CSU suggests that we advance from a focus upon endotypes towards a paradigm of predicting individual response.

## Author Contributions

N.C., A.D.I., K.R.: conceptualisation. C.O'F., N.C.: funding acquisition. K.R., R.A.: visualisation, writing – original draft preparation. B.M., C.O'F., J.D., C.M.F., A.D.I., N.C.: writing – review and editing. N.C., A.D.I., C.O'F., C.M.F., B.M., J.D.: supervision.

## Conflicts of Interest

N.C. has received support for attending meetings from Novartis. A.D.I. is a consultant/on the advisory board for AbbVie, Novartis, Regeneron, Sanofi, Leo Pharma, Pfizer, Eli Lilly, Benevolent AI and Arena. The remaining authors declare no conflicts of interest.

## Data Availability

Data sharing is not applicable to this article as no new data were created or analyzed in this study.
